# Sputum from People with Cystic Fibrosis Reduces the Killing of Methicillin-Resistant *Staphylococcus aureus* by Neutrophils and Diminishes Phagosomal Production of Reactive Oxygen Species

**DOI:** 10.3390/pathogens12091148

**Published:** 2023-09-09

**Authors:** Kayla M. Fantone, Joanna B. Goldberg, Arlene A. Stecenko, Balázs Rada

**Affiliations:** 1 Department of Infectious Diseases, College of Veterinary Medicine, The University of Georgia, Athens, GA 30602, USA; kayla.fantone25@uga.edu; 2 Division of Pulmonology, Asthma, Cystic Fibrosis and Sleep, Department of Pediatrics, Emory University School of Medicine, Atlanta, GA 30602, USA; joanna.goldberg@emory.edu (J.B.G.); astecen@emory.edu (A.A.S.)

**Keywords:** cystic fibrosis, PMN, MRSA, phagocytosis, sputum, phagosome, respiratory burst

## Abstract

Cystic fibrosis (CF) airway disease is characterized by chronic polymicrobial infections and an infiltration of neutrophils (PMNs). *Staphylococcus aureus* has been the most prevalent respiratory pathogen in CF. In particular, methicillin-resistant *S. aureus* (MRSA) represents a huge clinical burden in CF due to its association with lung disease and increased resistance to antibiotics. In CF, PMNs are unable to kill and clear MRSA. The reason for this remains largely unknown. Our study found that CF PMNs are as equally capable of killing MRSA as healthy PMNs. We show that the CF sputum, however, significantly impairs the ability of human PMNs to kill CF MRSA isolates. In the absence of CF sputum, PMNs kill MRSA via intracellular mechanisms mediated by phagocytosis, rather than extracellular mechanisms via NET formation. CF sputum does not affect the phagocytosis of MRSA via healthy or CF PMNs. Our results demonstrate that CF sputum exposure impairs phagosomal levels of reactive oxygen species (ROS) in MRSA-phagocytosing PMNs. While phagosomal co-localizations of MRSA with primary granule markers, myeloperoxidase and cathepsin D, were significantly reduced upon CF sputum exposure, that of a third azurophilic granule marker, neutrophil elastase, remained unaffected. This suggests that CF sputum does not compromise the fusion of primary granules with phagosomes but diminishes phagosomal ROS levels via another, likely more specific, mechanism. Overall, we identified the airway environment as an important factor that restricts neutrophils’ oxidative microbicidal activities in CF against MRSA. These results deliver new details of the complex host–pathogen interactions present in the CF lung.

## 1. Introduction

Cystic fibrosis (CF) is a life-threatening, inherited disease that affects the functioning of the airway epithelium causing severe damage and inflammation in the lungs. Lung disease in CF patients is characterized by reduced mucociliary clearance, chronic inflammation and polymicrobial infections starting in infancy [1,2]. Lung disease is the leading cause of morbidity and mortality in people with CF (PwCF) due to the progression of chronic respiratory infections and inflammation dominated by polymorphonuclear neutrophilic granulocytes (PMNs) [3,4]. PMNs are crucial for the clearance of bacterial pathogens through various mechanisms. However, despite the high numbers of PMNs found in the CF lung, they are unable to kill particular bacterial pathogens, and infections persist.

According to the United States’ Cystic Fibrosis Foundation’s annual report in 2021, *Staphylococcus aureus* (*S. aureus*) remains the most prevalent pathogen in CF, infecting 64% of PwCF, whereas only 28% were infected with *P. aeruginosa* [5]. Antibiotic resistance is on the rise and methicillin-resistant *S. aureus* (MRSA) contributes to many chronic *S. aureus* infections in PwCF [5]. About 30–40% of PwCF under the age of 30 years that have tested positive for *S. aureus* in their respiratory cultures are infected with MRSA and this number rises to about 50% or more in those over the age of 30 years [5,6]. The average age of PwCF when MRSA was first isolated from their airways is around 11 years [7]. The presence of MRSA in respiratory cultures from PwCF is associated with worse lung function, and the probabilities of hospitalization and treatment with oral, inhaled, and intravenous antibiotics have all been significantly higher in PwCF with MRSA compared to those infected with methicillin-sensitive *S. aureus* (MSSA) [8]. The prevalence of MRSA among CF patients has not decreased with the widespread use of highly effective modulators [9]. Therefore, MRSA is a prevalent pathogen in PwCF throughout their lifetime and tolerance to antibiotics contributes to the difficulty in finding treatment [6].

PMNs are the main drivers in killing pathogens such as *S. aureus.* However, they are inefficient in doing so in the CF lung. *S. aureus* is an opportunistic pathogen, and our previous research demonstrates that PMNs isolated from the blood of healthy individuals and PwCF are capable of killing CF clinical isolates of *S. aureus* including MRSA [10]. However, once healthy PMNs are exposed to CF sputum, they are less capable of killing CF clinical isolates of *S. aureus*, including both MSSA and MRSA [10]. Therefore, our goal was to understand how PMNs become ineffective in killing MRSA in the CF airways. Due to its clinical relevance, we focused our studies on MRSA. We previously described that CF sputum does not negatively affect the extracellular killing of *S. aureus* through NETosis [10]. *S. aureus* is predominantly killed by PMNs through phagosomal ROS generation following phagocytosis. Therefore, our goal was to further investigate this mechanism and understand how CF sputum affects interactions of phagosomes with MRSA during infection of PMNs [11,12,13]. The final stages of phagosomal maturation include phagolysosome fusion where granules containing antimicrobial enzymes such as neutrophil elastase (NE), myeloperoxidase (MPO), and cathepsin D (CatD) are released into the phagosome to target and kill pathogens. Therefore, we hypothesized that the CF airway environment impairs phagosomal maturation in PMNs during MRSA infection that leads to impaired bacterial killing.

## 2. Materials and Methods

### 2.1. Human Subjects

All the human subject studies were performed by following the guidelines of the World Medical Association’s Declaration of Helsinki.

### 2.2. Healthy Subjects

Healthy human subjects were recruited at the Clinical and Translational Research Unit (CTRU) at the University of Georgia (UGA) and provided informed consent before blood donation for PMN isolation according to the protocol UGA# 2012-10769-06. Healthy subjects were chosen to match the sex and age distributions of the CF subjects. Healthy subjects were defined as individuals that did not suffer from CF or an autoimmune disease based on a self-report.

### 2.3. People with CF

CF subjects were recruited from the Adult CF Clinic at Emory University. PwCF signed informed consent to provide blood and sputum samples (IRB00042577). CF diagnosis was confirmed via pilocarpine iontophoresis sweat testing and/or CFTR gene mutation analysis showing the presence of two disease-causing mutations. Sputum cultures were taken on the day of the clinic visit when the blood was drawn, and the presence or absence of bacterial infection was identified by the clinical microbiology laboratory. Baseline lung function was defined according to the guidelines of the CF Foundation Patient Registry, which is the average of the best percent predicted forced expired volume in one second (FEV_1_) for each quarter of the calendar year. Blood was drawn into EDTA-coated tubes and one silicone-coated tube, and processed the same day for PMN isolation and serum collection.

### 2.4. Neutrophil and Serum Isolation

PMNs and serum were isolated from the peripheral blood of healthy donors or PwCF. Of the blood, 20–40 milliliters were drawn into EDTA-coated tubes, and PMNs were isolated using the EasySep™ Direct Human PMN Isolation Kit (Catalog #19666, Stem Cell technologies, Vancouver, BC, Canada) according to the manufacturer’s protocol. This protocol uses the negative selection of neutrophils and routinely yields 30–130 × 10^6^ live PMNs with >99% viability and ~98% purity. An additional ten milliliters of blood were drawn into a separate, silicone-coated tube without anticoagulant and allowed to clot at room temperature for 30 min. The tube was centrifuged twice (1300× *g*, 5 min), the resulting supernatant (serum) was collected, and the coagulant was discarded. The serum was kept on ice for same-day experiments or frozen at −80 °C for future work.

### 2.5. CF Sputum Collection and Processing

All human studies involving sputum collection from PwCF were approved by the Emory University Institutional Review Board and were in accordance with institutional guidelines. All donors gave consent before sputum collection. Sputum samples were processed and modified using the previously published method [10,14]. CF sputum cocktails were made as previously published [10]. Briefly, sputum supernatants from five PwCF were pooled and PMNs were exposed to 30% (*v/v*) CF sputum cocktail for 3.5 h at 37 °C, according to our previously established protocol of this “CF sputum model” [15]. Following incubation, PMNs were washed twice with 1 × HBSS and resuspended in an “assay medium” (1 × HBSS, 10 mM HEPES, 5 mM glucose, and 1% (*v/v*) autologous serum). The CF sputum cocktail was also diluted in an “assay medium” to reach the desired 30% concentration.

### 2.6. Bacteria

Four methicillin-resistant *S. aureus* (MRSA) isolates recovered from the respiratory tract of adults with CF were obtained from the Emory + Children’s Cystic Fibrosis Biospecimen Repository (CFBR). The age of the four PwCF who donated their MRSA ranged from 20 to 53 years. USA300 was used as a reference strain [16,17]. USA300 is a MRSA isolate derived from an adolescent patient with severe sepsis syndrome at the Texas Children’s Hospital. Table 1 lists the isolates used in this study that have also been described in detail in prior works [18,19].

For all experiments, MRSA isolates were grown on blood agar (TSA II 5% SB) at 37 °C overnight, and cultured the following day in an optimal growth medium (3 mL LB medium) at 37 °C with shaking for 2−3 h. The bacteria were collected and resuspended in 1 × HBSS, and the optical density was measured in a 96-well microplate at 600 nM in a Varioksan flash™ microplate spectrophotometer (Thermo Fisher Scientific, Waltham, MA, USA). As previously published, an optical density value of 0.6 was determined to correspond to 1.0 × 10^9^ CFU/mL bacterial concentration. For all experiments, bacteria were opsonized with 10% (*v/v*) autologous serum of the PMN donor (healthy control or PwCF) for 20 min at 37 °C. Following opsonization, the bacteria were washed via centrifugation and resuspended in an assay medium prior to infecting PMNs.

### 2.7. High-Throughput Bacterial Killing Assay

To assess bacterial killing by human PMNs, a high-throughput 384-well microplate-based assay was used based on our previously published method [10]. Briefly, purified human PMNs from PwCF and age- and gender-matched healthy controls were either left untreated or treated with a CF sputum cocktail, as described above. Following pretreatment of PMNs with or without CF sputum, opsonized bacteria were mixed at a ratio of 1:1 multiplicity of infection (MOI, MRSA: PMN) in microcentrifuge tubes. The tubes were incubated at 37 °C for 30 min, with mixing every 5–7 min. After 30 min, PMNs were lysed using 1 mg/mL saponin in 1 × HBSS on ice for 5 min and diluted in 1 × HBSS 100-fold. The saponin treatment alone did not affect MRSA viability while the alternative method using alkaline distilled water to burst PMNs [20] did kill MRSA in our hands. The samples were transferred to a 384-well microplate containing an LB broth at a 100-fold dilution in quadruplicates. The microplate was read in a microplate spectrophotometer Biotek Epoch 2™ (Agilent, Santa Clara, CA, USA). Bacterial growth was measured with an absorbance reading at 600 nm every 4 min for 16 h, with constant heating at 37 °C and shaking. After the measurement, growth curves were generated and bacterial killing was expressed as a decrease in surviving bacteria over time.

### 2.8. Drug Treatment of PMNs for Inhibition of Phagocytosis or NETs

To assess the primary mechanism for MRSA killing by PMNs from healthy donors, phagocytosis of PMNs was inhibited by 10 µM cytochalasin-B (MedChem Express, cat#14930-96-2) and NETs released from PMNs were degraded by 10 µg/mL DNAse I (Sigma-Aldrich, St. Louis, MO, USA, cat#AMPD1-1KT). After PMN isolation from the blood of healthy donors, PMNs were either untreated, pretreated with 10 μM cytochalasin-B, or 10 μg/mL DNAse I for 20 min at 37 °C. Following pretreatment, PMNs were infected with opsonized MRSA isolates as described above. The control samples were added with bacteria and drug alone (no PMNs), to assess whether drug affects bacterial growth itself.

### 2.9. Phagocytosis

Phagocytosis of MRSA24 by PMNs from healthy donors with or without the CF sputum cocktail treatment was determined by the imaging cytometer, Amnis^®^ ImageStream^®^-X Mark II™ (Millipore Sigma, Burlington, MA, USA) at the University of Georgia Coverdell Imaging Core Facility. Phagocytosis of MRSA24 by PMNs from healthy donors and CF donors, and from CF sputum-treated CF PMNs was performed by flow cytometry on the Novocyte Quanteon 4025™ (Aligent, Santa Clara, CA, USA). MRSA24 was first stained with 5 mM pHrodo™ iFL green STP Ester (Invitrogen, cat#P35369) for 45 min at 37 °C, protected from light. After staining, pHrodo-stained MRSA24 was opsonized with 10% autologous serum for 15 min and added to PMNs at MOI of 1. PMN–MRSA24 cocultures were incubated for 1 h at 37 °C, protected from light. For negative control, PMNs were placed on ice and protected from light after the addition of pHrodo-stained MRSA24. After 1 h, PMNs were washed with 1 × PBS and resuspended in 1 × PBS.

For ImageStream^®^ analysis version 6.2 (v.6.2) (Cytek Biosciences, Fremon CA, USA), PMNs were fixed with a stabilizing fixative (BD Biosciences, cat#338306) at a 1:3 dilution for 15 min. After fixation, PMNs were washed and resuspended with 1 × PBS, and stained with 4’,6-diamidino-2-phenylindole (DAPI) nucleic acid stain (Sigma-Aldrich, cat#28718-90-3) at 300 nM for 15 min at room temperature. After DAPI staining, PMNs were washed and resuspended in 100 µL 1 × PBS and analyzed using ImageStream^®^ v.6.2 at a 40× resolution. For MRSA24 (pHrodo green) detection, a blue laser at 488 nm was used and read at channel 2 with the 480–560 nm filter. For DAPI detection, the violet laser at 405 nm was used and read at channel 7 with the 430–505 nm filter. Data were analyzed with the IDEAS^®^ v.6.2 ImageStream^®^ software at the University of Georgia Coverdell Biomedical Microscopy Core.

For flow cytometry analysis of phagocytosis of MRSA24, PMNs were stained with Zombie Aqua™ Fixable Viability kit (Biolegend, San Diego, CA, USA cat#423102) at a dilution of 1:500 for 15 min after washing following infection. PMNs were then washed and resuspended in 1% BSA in 1 × PBS. PMNs were stained with the granulocyte marker, CD66b, AlexaFluor 647 (Biolegend, cat#561645), at a final concentration of 1 μg/mL for 30 min. PMNs were then washed and resuspended in a BD-stabilizing fixative and analyzed on the Novocyte Quanteon™. Zombie Aqua fluorescence was detected by a violet laser at 405 nm with the 525/50 filter, MRSA24 (pHrodo-green) was detected by a blue laser at 488 nm with the 530/30 filter, and CD66b was detected by a red laser at 637 nm with the 660/20 filter. Data were analyzed at the University of Georgia College of Veterinary Medicine Cytometry Core Facility with the NovoSamplerQ utilizing NovoExpress software v.1.4.1.

### 2.10. Neutrophil Elastase Activity Assay

To measure neutrophil elastase (NE) activity, PMNs were either untreated or treated with a CF sputum cocktail, as described above. Following pretreatment, PMNs were either placed on ice or infected with MRSA24 at MOI of 1 for 30 min at 37 °C. For a positive control, 2 µM of the PMN stimulator, N-formylmethionyl-leucyl-phenylalanine was added. Following infection, uninfected and infected PMNs were centrifuged at 450 g for 5 min at 4 °C to pellet PMNs. The supernatant was transferred to new microcentrifuge tubes and kept at 4 °C. The PMN cell pellet was resuspended in 100 µL of 1 × PBS and sonicated for 30 s to lyse the cells. After cell lysis, PMNs were centrifuged at 10,000 g for 5 min at 4 °C to remove cell debris. The supernatant was collected into new microcentrifuge tubes and labeled as lysates. The NE activity of these samples was determined by following the manufacturer’s protocol for the fluorometric neutrophil elastase activity assay kit (Cayman Chemical, cat#600610). A standard curve was generated with human NE diluted in a cell assay buffer ranging from a concentration of 0.156 to 10.0 mU/mL. A substrate solution diluted in dimethylformamide was added to the samples, standards, and blanks. Enzymatic activity was detected with a fluorometric microplate reader, Varioskan Flash™ (Thermo Scientific, Waltham, MA, USA) at an excitation wavelength of 485 nm and an emission wavelength of 525 nm. Fluorescence kinetics were measured every 2 min for 2 h at 37 °C. The average relative fluorescence units (RFUs) were read and the NE activities (mU/mL) of the samples were calculated using the standard curve equation generated.

### 2.11. Phagolysosome Fusion via ImageStream Cytometry

To assess the co-localization of NE, MPO, and CatD with MRSA24 (as a measure of phagolysosome fusion between *S. aureus*-containing phagosomes and primary granules), ImageStream^®^ analysis was performed. PMNs were isolated from healthy donors and/or PwCF, and either untreated or treated with a CF sputum cocktail as described above. MRSA24 was stained with pHrodo green as previously described, and PMNs were infected with pHrodo-stained MRSA24 for 30 min at 37 °C at an MOI of 1, protected from light. For a negative control, pHrodo-stained MRSA24 was added to PMNs and immediately placed on ice, protected from light. Following infection, PMNs were washed and resuspended in a BD-stabilizing fixative at a 1:3 dilution for 15 min at room temperature. After fixation, PMNs were washed with 1 × PBS and resuspended in a blocking buffer (5% BSA in 1 × PBS containing 10% horse serum and 0.1% Triton-X100). PMNs were blocked for 30 min at room temperature, protected from light. After blocking, PMNs were washed with 5% bovine serum albumin (BSA) and resuspended in 1% BSA in 1 × PBS with 5% horse serum. Primary antibodies were added at a 1:500 dilution overnight at 4 °C. The antibodies used were anti-NE rabbit polyclonal antibody (Thermo Scientific, cat#PA5-87158), anti-MPO rabbit polyclonal antibody, unconjugated (Thermo Scientific, cat#PA5-16672), and anti-cathepsin D rabbit polyclonal antibody (Proteintech, cat#2137-1-AP). The next day, PMNs were washed with 1% BSA in 1 × PBS and resuspended in 1% BSA with 1% horse serum and stained with secondary antibody for 1 h at room temperature. The secondary antibody used was VectaFluor™ horse anti-rabbit IgG, Dylight^®^ 594 antibody at a 1:200 dilution. Following incubation with the secondary antibody, PMNs were washed with 1% BSA and resuspended in 1 × PBS and stained with 300 nM DAPI nuclear stain for 15 min at room temperature. Following DAPI stain, PMNs were washed and resuspended in 100µL 1 × PBS and quantified using ImageStream^®^ at a 40× resolution. For MRSA24 (pHrodo green) detection, a blue laser at 488 nm was used and read at channel 2 with the 480–560 nm filter. For NE, MPO, and CatD detection, a yellow laser at 561 nm was used and read at channel 4 with the 595–640 nm filter. For DAPI, a violet laser at 405 nm was used and read at channel 7 with the 430–505 nm filter. The data were analyzed with the IDEAS^®^ v.6.2 ImageStream^®^ software with the colocalization wizard.

### 2.12. Myeloperoxidase Activity Assay

Myeloperoxidase (MPO) activity was measured with the Myeloperoxidase Chlorination Fluorometric Assay Kit (Cayman Chemical, cat#10006438), following the manufacturer’s protocol. PMNs were prepared as described above in the neutrophil elastase activity assay. A standard curve was generated with the fluorescein standard diluted in an MPO assay buffer ranging from 5 nM to 200 nM. An initiator solution was added to the samples, standards, and blanks, which consisted of the MPO assay buffer with 16.4% chlorination substrate, and 8.2% of 5 mM hydrogen peroxide. The detection of chlorination was carried out using a fluorometric microplate reader, Varioskan Flash™, at an excitation wavelength of 485 nm and an emission wavelength of 520 nm. Fluorescence kinetics were measured every min for 1 h. The average RFUs were taken for all samples and standards, and the MPO activity (nM) of each sample was calculated using the standard curve equation generated.

### 2.13. Phagosomal ROS Assay via ImageStream Cytometry

To measure phagosomal ROS production by PMNs after exposure to the CF sputum cocktail, ImageStream^®^ analysis was performed. For all experiments, PMNs were immediately placed on ice after administration of MRSA24 and/or OxyBURST^TM^ for the negative control.

To measure total intracellular ROS production, PMNs were isolated from healthy donors and either untreated or treated with a CF sputum cocktail, as previously described. Following sputum exposure and washing, PMNs were given 10 µM of OxyBURST™ Green H2DCFDA, SE (Invitrogen, cat#D2935), for 1 h at 37 °C. After 1 h, PMNs were washed with 1 × PBS and resuspended in 100 µL of 1 × PBS and placed on ice. Live PMNs were analyzed using ImageStream^®^. For the detection of OxyBURST-positive PMNs, samples were analyzed at a 40× resolution and a green laser at 488 nm was used with channel 2 (480–560 nm filter).

To measure the co-localization of OxyBURST and MRSA24 as indicators of phagosomal ROS production, PMNs were isolated from healthy donors and either left untreated or treated with a CF sputum cocktail. MRSA24 was stained with 5 mM pHrodo™ Red, succinimidyl ester (Invitrogen, cat#P36600) for 45 min at 37 °C. After pHrodo Red staining, MRSA24 was washed with 1 × HBSS and also stained with 1 mM of OxyBURST for 45 min at 37 °C. Following the OxyBURST stain, pHrodo-oxyBURST-stained MRSA24 was opsonized with 10% (*v/v*) autologous serum for 15 min at 37 °C. pHrodo-oxyBURST-stained MRSA24 was washed and resuspended in assay media and added to PMNs at an MOI of 1 for 1 h at 37 °C. After 1 h, PMNs were washed, resuspended in 1 × PBS, and live PMNs were analyzed using ImageStream^®^ at a 40× resolution. Since OxyBURST was only present on the surface of the bacterium, OxyBURST fluorescence was indicative of ROS production near the bacterial surface. When MRSA-bound OxyBURST co-localized with MPO in PMNs, it detected phagosomal ROS production.

### 2.14. Statistical Analysis

The results between PMNs treated with and without CF sputum were analyzed using the two-tailed, paired Student’s *t*-test. The data between CF and healthy PMNs were analyzed using the Mann–Whitney test. Data are expressed as the mean plus–minus the standard error of mean (SEM). Statistically significant differences were considered as *, *p* < 0.05; **, *p* < 0.01; ***, *p* < 0.001; ^ns^, not significant. Statistical analyses were carried out with GraphPad Prism version 9.0 for Windows software (GraphPad Software, San Diego, CA, USA).

## 3. Results

### 3.1. CF Sputum Treatment Impairs CF PMNs’ Ability to Kill MRSA

**Figure 1:**
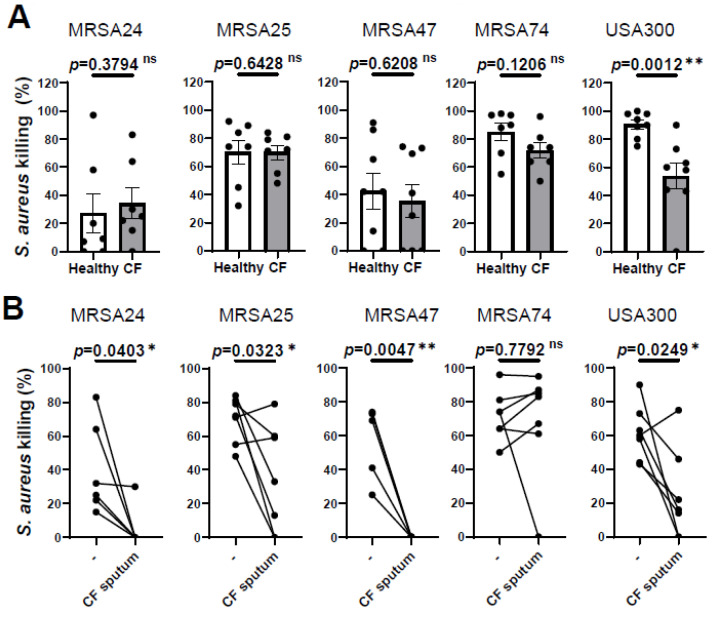
CF sputum treatment impairs the ability of CF PMNs to kill MRSA. (**A**) PMNs were isolated from the blood of PwCF or healthy donors and infected with each of the indicated four MRSA clinical isolates and USA300 (n = 7–8). Bacterial killing was measured via a high-throughput microplate-based assay. Mean ± SEM. The data were analyzed using the Mann–Whitney test. (**B**) CF PMNs were isolated from the blood of PwCF, and were left either untreated or treated with 30% (*v/v*) CF sputum supernatant cocktail and then infected with each of the indicated four MRSA isolates and or USA300 at an MOI of 1 for 30 min (n = 4–7). Control CF PMNs were treated with equal amount of “assay medium” (-). Paired two-tailed Student’s *t*-test. Statistically significant differences were considered ns, not significant; *, *p* < 0.05; **, *p* < 0.01; CF, cystic fibrosis; MOI, multiplicity of infection.

Our previous results demonstrated that PMNs isolated from PwCF killed MRSA CF clinical isolates to the same extent as PMNs derived from healthy individuals [10] To further confirm this observation, we recruited additional PwCF and age- and sex-matched healthy donors, and obtained the same result (Figure 1A). Additionally, we noticed that PMN-mediated killing of only the reference strain, USA300, was impaired in PwCF (Figure 1A). Thus, we provide evidence that CF PMNs are capable of killing CF clinical isolates of MRSA in vitro.

We have also shown previously that CF sputum impaired the ability of PMNs isolated from healthy donors to kill CF clinical isolates of MRSA [10]. Next, we explored whether CF sputum had similar effects on PMNs isolated from the blood of PwCF, as well. PMNs from PwCF were first exposed to the CF sputum cocktail and then infected with MRSA, according to our standard protocol. The CF sputum and CF PMNs were not patient-matched. CF sputum supernatants were pooled from five PwCF who differed from the CF PMN donor. Our results show that MRSA killing was significantly reduced when CF PMNs were exposed to the CF sputum for three out of the four clinical isolates, as well as the lab reference strain USA300 (Figure 1B). CF PMN-mediated killing of MRSA74 was not affected by the CF sputum at all (Figure 1B). These data are consistent with our prior results obtained on healthy PMNs (7). Overall, we observed that CF PMNs are not compromised in killing MRSA in vitro and CF sputum reduces the ability of PMNs from both healthy individuals and PwCF to kill MRSA.

### 3.2. MRSA Killing by PMNs Is Predominantly Mediated by Phagocytosis, Not NET Release

**Figure 2:**
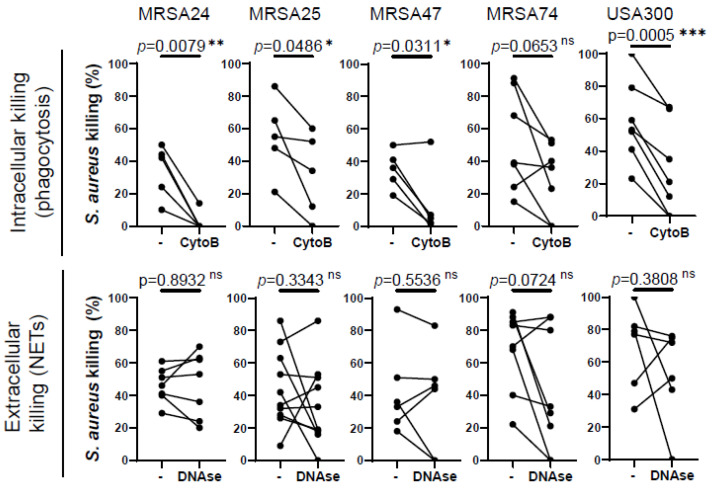
Phagocytosis is required for MRSA killing by human PMNs. PMNs were isolated from healthy donors, and were left untreated or treated with (**top**) 10 µM cytochalasin-B (CytoB) (n = 5–7) or (**bottom**) 10 U/mL of DNase (n = 5–7) for 30 min and infected with four MRSA clinical isolates and the lab reference strain (USA300) at an MOI of 1 for 30 min. Bacterial killing was measured via a high-throughput microplate-based assay. The control PMNs (“-“) were treated with equal amounts of solvents (DMSO or “assay medium”). Mean ± SEM. The data were analyzed using the two-tailed paired Student’s *t*-test. Statistically significant differences were considered *, *p* < 0.05; **, *p* < 0.01; ***, *p* < 0.001. NET, neutrophil extracellular traps; ns, not significant; CytoB, cytochalasin-B; MOI, multiplicity of infection.

Our next goal was to identify how MRSA is predominantly killed by healthy PMNs. PMNs kill microorganisms through two major mechanisms, intracellular killing following phagocytosis and extracellular killing mediated by NETs. To determine which mechanism human PMNs use to kill CF clinical isolates of MRSA in our experimental system, phagocytosis was inhibited by the addition of cytochalasin-B (which blocks the formation of contractile microfilaments and inhibits PMN phagocytosis of bacteria [21,22], while the formed NETs were destroyed by treatment with DNase I. Our data demonstrate that the killing of three out of the four MRSA clinical isolates is significantly impaired when phagocytosis was inhibited in healthy PMNs (Figure 2). *S. aureus* CF clinical isolates of which the killing was impaired by cytochalasin-B treatment correspond to the same isolates of which the killing by PMNs was compromised by the CF sputum, suggesting that CF sputum impairs intracellular bacterial killing. MRSA74 killing by PMNs was not impaired by the CF sputum or by inhibition of phagocytosis (Figure 2). The killing of none of the MRSA CF isolates was inhibited when NETs were degraded by the addition of DNase, although NETs may play some role in killing MRSA74, as the *p*-value was 0.0724 (Figure 2). Additionally, healthy PMNs were significantly impaired in killing USA300 when phagocytosis was inhibited, but not when NETs were destroyed (Figure 2). Overall, we propose that MRSA is predominantly killed in healthy PMNs via phagocytosis-initiated intracellular mechanisms.

### 3.3. CF Sputum Does Not Diminish S. aureus Phagocytosis by CF PMNs

**Figure 3:**
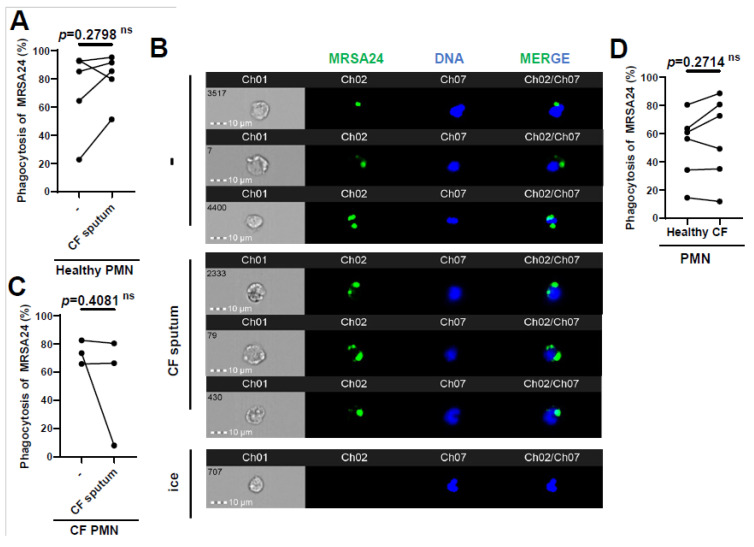
CF sputum does not inhibit MRSA phagocytosis by healthy or CF PMNs. (**A**) PMNs from healthy donors were isolated, and were either untreated or treated with CF sputum and infected with pHrodo-stained MRSA24 for 1 h. PMNs were placed on ice as a negative control. Phagocytosis was measured via ImageStream cytometry (n = 5). (**B**) Representative images of (**A**), DNA (blue), MRSA24 (green). The data were analyzed using the two-tailed paired Student’s *t*-test. (**C**) Phagocytosis of pHrodo-stained MRSA24 from PMNs isolated from CF subjects and either untreated or treated with CF sputum, measured via ImageStream cytometry (n = 3). (**D**) PMNs were isolated from healthy donors or CF subjects and infected with pHrodo-stained MRSA24, and phagocytosis was measured via flow cytometry (n = 6). Mean ± SEM. The data were analyzed using the Mann–Whitney test. Statistically significant differences were considered as. CF, cystic fibrosis; PMN, neutrophil; ns, not significant.

Since we observed that phagocytosis is the main mechanism by which healthy PMNs kill MRSA, we measured whether phagocytosis was affected in CF PMNs or by CF sputum exposure. Opsonized MRSA24 was labeled with pHrodo (green) and fed to human PMNs for 30 min. Phagocytosed bacteria were visualized via imaging flow cytometry analysis (Figure 3, PMN DNA is indicated in blue). We demonstrated that healthy PMNs exposed to CF sputum were not impaired in phagocytosing MRSA24 (Figure 3A,B). Similarly, the CF sputum did not inhibit CF PMNs’ ability to phagocytose MRSA24 (Figure 3C). Additionally, there was no significant difference in the phagocytosis of MRSA24 by PMNs from healthy donors vs. PwCF (in the absence of CF sputum) (Figure 3D). Our results suggest that the impairment of PMN-mediated MRSA killing by CF sputum is not due to an inhibition of phagocytosis and that CFTR deficiency does not lead to reduced phagocytic abilities in human PMNs.

### 3.4. CF Sputum Impairs Phagosomal ROS Levels in MRSA-Phagocytosing Human PMNs

**Figure 4:**
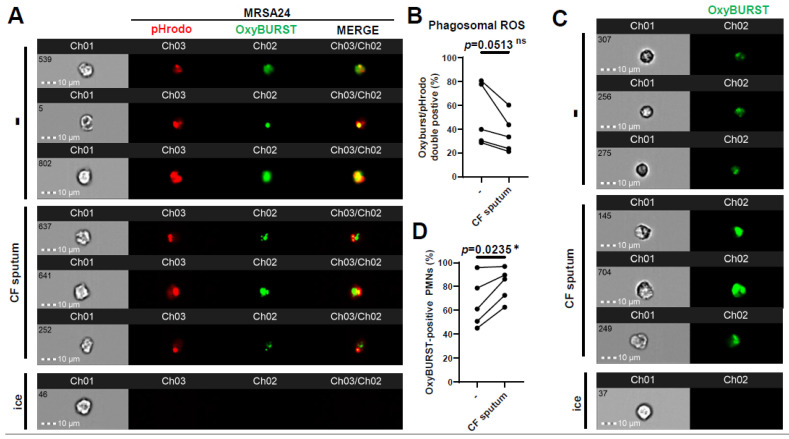
CF sputum impairs phagosomal ROS production in MRSA-phagocytosing human PMNs. PMNs were isolated from healthy donors and were either left untreated or treated with CF sputum in our “CF sputum model”. The PMNs placed on ice served as the negative control. (**A**) MRSA24 was stained with pHrodo-red for 45 min, then with OxyBurst green for 45 min prior to infection. PMNs were infected with pHrodo-OxyBurst-stained MRSA24 following CF sputum treatment for 1 h at an MOI of 1. Representative images are shown: pHrodo (red), and OxyBurst (green). (**B**) The PMNs double-positive for OxyBurst and pHrodo were analyzed via ImageStream cytometry (n = 5). (**C**) Following CF sputum treatment of PMNs, 10 µM of OxyBurst green was added for 1 h. Representative images are shown: OxyBurst (green). (**D**) OxyBurst-positive cells were analyzed via ImageStream cytometry (n = 5). Mean ± SEM. The data were analyzed using the two-tailed paired Student’s *t*-test. Statistically significant differences were considered *, *p* < 0.05. CF, cystic fibrosis; ROS, reactive oxygen species; ns, not significant.

ROS represent an important component of PMNs for killing *S. aureus.* Therefore, we investigated whether phagosomal ROS production was impaired by the CF sputum. This was carried out by surface-labeling MRSA24 with two fluorescent stains: pHrodo to detect *S. aureus* in the phagosome, and OxyBurst to detect H_2_O_2_ production close to the bacterial surface. Healthy PMNs were left untreated or treated with the CF sputum, followed by the addition of opsonized MRSA24 labeled with pHrodo (green). Co-localization of pHrodo with Oxyburst identifies ROS produced in MRSA-containing PMN phagosomes. Interestingly, CF sputum inhibited phagosomal ROS production in MRSA24-phagocytosing human PMNs (Figure 4A,B). To explore how CF sputum treatment affected the total intracellular ROS output, the effect of CF sputum on OxyBurst-labeled human PMNs was measured. Surprisingly, a significant increase in total ROS output was observed (Figure 4C,D). The impairment of phagosomal ROS production is not a consequence of reduced total ROS production. These results suggest that CF sputum limits the phagosomal generation of ROS to MRSA24 that likely impairs subsequent phagosomal bacterial killing.

### 3.5. CF Sputum Reduces the Co-Localization of MPO with Phagocytosed MRSA

**Figure 5:**
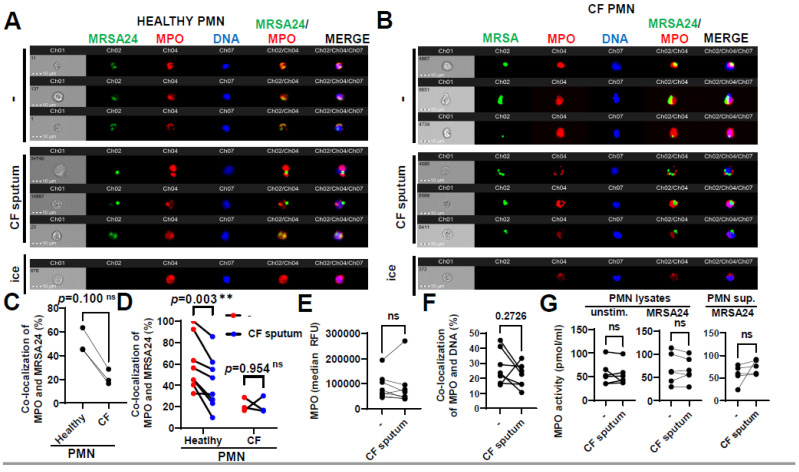
CF sputum reduces the co-localization of MPO with phagocytosed MRSA. (**A**) PMNs were isolated from healthy donors and were left either untreated or treated with CF sputum. PMNs then were infected with pHrodo green-stained MRSA24 for 30 min at an MOI of 1. PMNs were then fixed and stained with an MPO antibody. Neutrophils were placed on ice as a negative control. Representative images are shown obtained via ImageStream: MRSA (green), MPO (red), DNA (blue). (**B**) PMNs were isolated from CF subjects, and were either untreated or treated with CF sputum and infected with pHrodo green-stained MRSA24 for 30 min at an MOI of 1. PMNs were then fixed and stained with an MPO antibody. Representative images are shown: MRSA24 (green), MPO (red), DNA (blue). (**C**) Co-localization of MRSA24 and MPO in PMNs from healthy donors and CF subjects not treated with CF sputum, performed via ImageStream cytometry analysis (n = 3). (**D**) Co-localization of MRSA24 and MPO in PMNs from healthy donors (n = 8) and CF subjects, (n = 3) untreated (red) and CF-sputum-treated (blue). Analysis was performed via ImageStream cytometry. The data were analyzed using the Mann–Whitney test. (**E**) Median fluorescence intensity of MPO in healthy PMNs left untreated or treated with CF sputum was measured via ImageStream cytometry (n = 8). (**F**) Co-localization of MPO and DNA in healthy PMNs either untreated or treated with CF sputum was measured via ImageStream cytometry (n = 8). (**G**) Healthy neutrophils were either untreated or treated with CF sputum. PMNs were then either lysed or infected with MRSA24 at an MOI of 1 for 30 min. After infection, PMN supernatants were collected or lysed. MPO activity was measured using a fluorometric quantification (n = 5–6). Mean ± SEM. The data were analyzed using the two-tailed paired Student’s *t*-test. **, *p* < 0.01; ns, not significant; MPO, myeloperoxidase; PMN, neutrophil.

Primary granules containing MPO fuse with the phagosome in *S. aureus*-engulfing PMNs; therefore, the effect of CF sputum on co-localization of MPO with MRSA was assessed. MPO trafficking to the phagosome was measured in PMNs obtained from healthy subjects (Figure 5A) or PwCF (Figure 5B) infected with MRSA24, in the absence of any CF sputum treatment. No significant difference was observed in MPO trafficking to MRSA-containing phagosomes between CF and healthy PMNs (Figure 5C). Interestingly, CF sputum pretreatment resulted in decrease in MPO co-localization with MRSA in healthy PMNs that was absent in CF PMNs (Figure 5D). CF sputum exposure did not affect MPO fluorescence values indicative of MPO protein levels per PMN (Figure 5E). This excludes the possibility that reduced co-localization of MPO with MRSA is a consequence of diminished MPO levels per PMN. MPO co-localization with DNA was not changed by the CF sputum (Figure 5F), indicating that hijacking MPO by DNA (to likely promote NET release) is not the reason for the concomitant decreased levels of MRSA-associated MPO.

To observe if CF sputum affects the enzymatic activity of MPO in PMNs, we determined MPO activity using an MPO chlorination assay. MPO activity in PMNs or in their supernatants remained unimpaired by the CF sputum, in the absence or presence of MRSA (Figure 5G).

### 3.6. CF Sputum Does Not Affect NE Co-Localization with Phagocytosed MRSA

**Figure 6:**
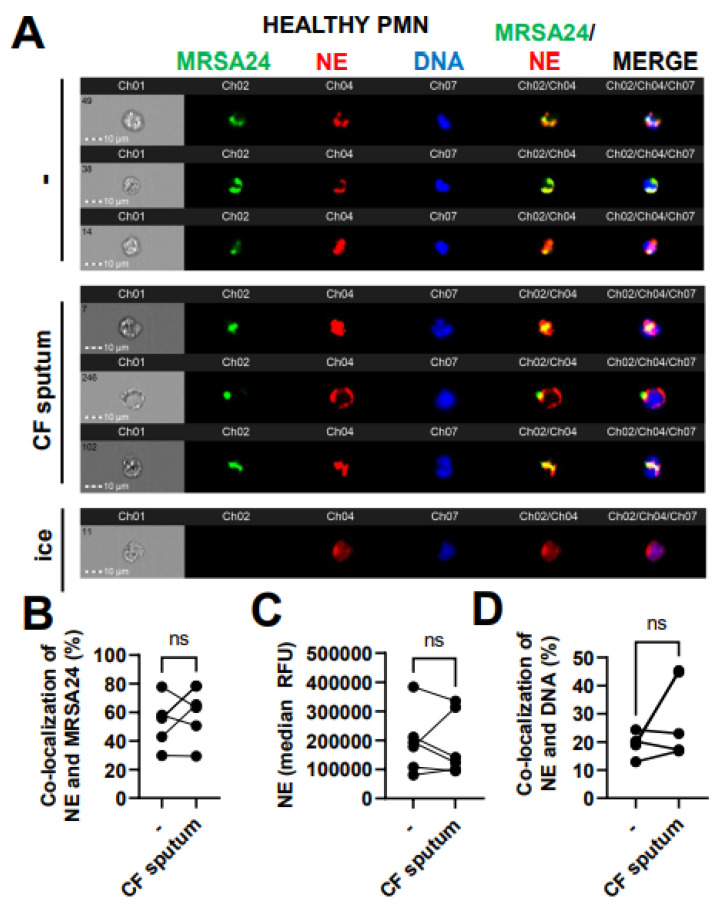
CF sputum does not affect neutrophil elastase co-localization with phagocytosed MRSA. (**A**) PMNs were isolated from healthy donors and were either left untreated or treated with CF sputum and infected with pHrodo green-stained MRSA24 for 30 min at an MOI of 1. PMNs were then fixed and stained with an NE antibody. PMNs placed on ice served as the negative control. Representative images are shown, performed via ImageStream: MRSA (green), NE (red), DNA (blue). (**B**) Co-localization of MRSA24 and NE in PMNs from healthy donors, untreated or treated with CF sputum, was performed via ImageStream cytometry analysis (n = 6). (**C**) The median fluorescence intensity of NE in healthy PMNs, untreated or treated with CF sputum, was measured via ImageStream cytometry (n = 6). (**D**) Co-localization of NE and DNA in healthy PMNs either untreated or treated with CF sputum were measured via ImageStream cytometry (n = 4). Mean ± SEM. The data were analyzed using the two-tailed paired Student’s *t*-test. NE, neutrophil elastase; ns, not significant.

Phagosomal maturation is a crucial step to ensure bacterial death inside the phagosome of PMNs. Reduced MPO–MRSA co-localization in PMNs by the CF sputum could be due to an impaired fusion of MPO-containing primary granules with the MRSA-containing phagosome. We aimed to determine whether CF sputum impairs phagolysosome fusion in MRSA-infected healthy PMNs using another primary granule marker, NE. The co-localization of NE with MRSA24 (present in the phagosome) was not affected by the CF sputum (Figure 6A,B). The total NE levels in PMNs were not affected by the CF sputum (Figure 6C). NE can also travel to the nucleus to mediate the process of NET formation [23]. The CF sputum does not impair NE trafficking to the nucleus in MRSA24-infected PMNs (Figure 6D). Altogether, the CF sputum does not affect NE/MRSA24 co-localization.

### 3.7. CF Sputum Reduces CatD Co-Localization with Phagocytosed MRSA

**Figure 7:**
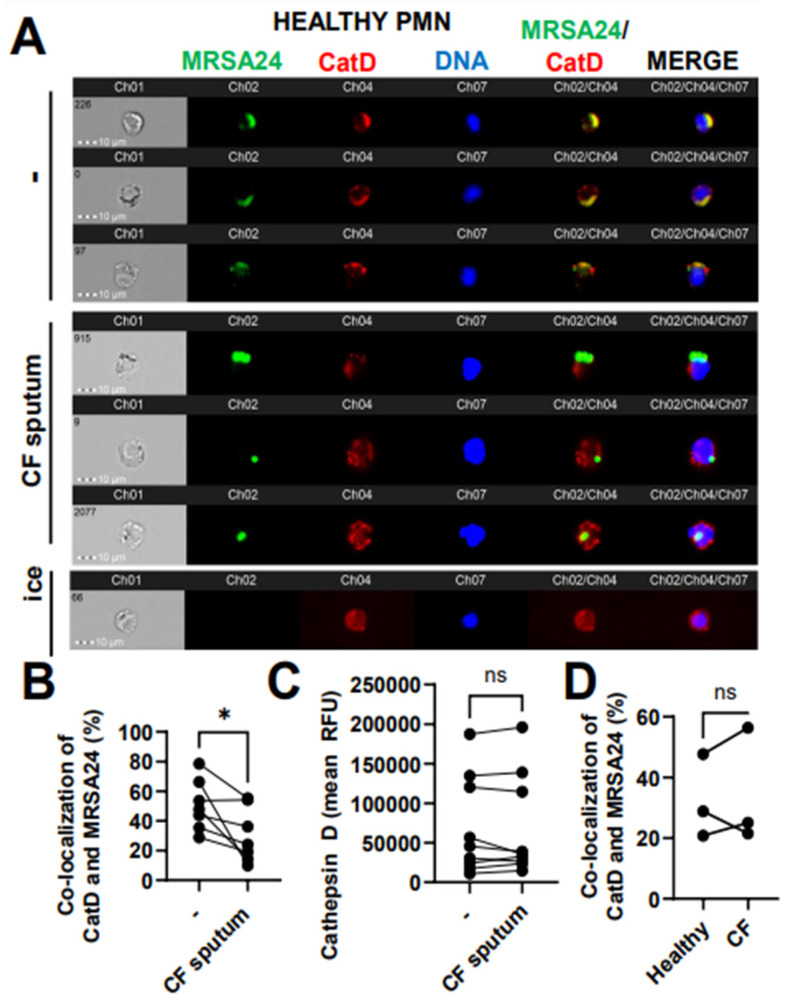
CF sputum reduces cathepsin D co-localization with phagocytosed MRSA. (**A**) PMNs were isolated from healthy donors and were either left untreated or treated with CF sputum. PMNs were then infected with pHrodo green-stained MRSA24 for 30 min at an MOI 1. PMNs were fixed and stained with a CatD antibody. PMNs placed on ice served as the negative control. Representative images are shown, performed via ImageStream: MRSA24 (green), CatD (red), and DNA (blue). (**B**) Co-localization of MRSA24 and CatD in PMNs from healthy donors, untreated or treated with CF sputum, was performed via ImageStream cytometry analysis (n = 7). (**C**) Median fluorescence intensity of CatD in healthy PMNs untreated or treated with CF sputum and measured via ImageStream cytometry (n = 7). (**D**) PMNs were isolated from healthy donors and CF subjects, and infected with pHrodo-stained MRSA24. Co-localization of CatD and MRSA24 was measured via ImageStream cytometry (n = 3). Mean ± SEM. The data were analyzed using the two-tailed paired Student’s *t*-test. Ns, not significant; *, *p* < 0.05; CatD, cathepsin D; RFU, relative fluorescent unit; CF; cystic fibrosis; MOI, multiplicity of infection.

To further explore the delivery of the primary granule content to MRSA24 in the phagosome, we measured the co-localization of MRSA with cathepsin D (CatD), a PMN aspartyl protease, a third-primary granule marker used in this study of which the release from the granules into the phagosome has been documented to be ROS-dependent. Healthy PMNs treated with CF sputum were significantly impaired in trafficking CatD into the phagosome during MRSA24 infection compared to untreated PMNs (Figure 7A, B). CatD levels in PMNs remained unaffected by the CF sputum (Figure 7C). No difference was observed in CatD/MRSA co-localization between healthy and CF PMNs (Figure 7D). Overall, we conclude that CF sputum impairs CatD trafficking to the phagosome in MRSA-infected PMNs.

## 4. Discussion

Our data demonstrate a unique mechanism to explain the impaired killing of MRSA by PMNs in the CF airway. Ultimately, we confirm that CF PMNs are largely capable of killing CF clinical isolates of MRSA, but exposure to the CF airway environment impairs PMN-mediated killing. In more than half of PwCF, lung disease is associated with persistent *S. aureus* infections, despite the abundance of PMNs in CF airways. This suggests that the CF airway environment impacts PMNs’ killing capabilities and we hypothesized that the CF airway environment impairs phagosomal maturation in MRSA-infected PMNs.

One of the earliest abnormalities in CF airway disease is the recruitment of PMNs to the lungs. PMNs’ primary killing mechanisms against pathogens including *S. aureus* involve intracellular killing through phagocytosis and extracellular killing by NETs [24,25]. While NETs are abundant in CF airways [26,27,28] and *S. aureus* induces NET release in human PMNs [29,30], *S. aureus* is fairly resistant to NET-mediated killing [29,31,32,33,34], suggesting that NETs released in the CF lung do not likely mediate elimination of *S. aureus*. *S. aureus* has been detected inside PMNs in CF airways, indicating that phagocytosis of *S. aureus* occurs to some extent in vivo [35]. Antibody- or complement-enhanced phagocytosis and the associated respiratory burst generating ROS represent the main mechanisms by which PMNs kill *S. aureus* [12,13,36].

One of the proposed mechanisms behind impaired bacterial killing of CF PMNs suggests that CFTR is expressed in healthy PMNs and plays an important role in supporting the phagocytic killing of microbes. CFTR is proposed to transport chloride into the phagosome for the formation of bactericidal HOCl and establishing an optimal ionic and pH environment [37,38]. In the PMNs of PwCF, this CFTR-mediated chloride transport would be deficient, and phagosomal bacterial killing would be compromised. CFTR gene expression and protein localization to secretory granules was reported in human PMNs [39]. Intraphagosomal chloride transport and the in vitro killing of the *P. aeruginosa* laboratory strain PAO1 have been shown to be impaired in human CF PMNs [39,40]. *Cftr*-deficient mice (global or myeloid-specific) exhibit defective clearance of laboratory strains or a mucoid clinical isolate of *P. aeruginosa* and suboptimal resolution of inflammation in an in vivo zymosan model [41,42,43]. While these data indicate that endogenous CFTR promotes bacterial killing in PMNs, the most significant differences were observed in mice that have several limitations for studying CF lung disease [44,45]. Only two manuscripts reported a somewhat impaired in vitro killing of *P. aeruginosa* [40] or *Burkholderia cenocepacia* [46] by human CF PMNs. There are no indications in the medical literature showing an impaired killing of *S. aureus* by human CF PMNs. On the contrary, we reported normal *S. aureus* killing in CF PMNs in vitro [10], and independent investigators published that the in vitro killing of *P. aeruginosa* by CF PMNs is not impaired [47]. To our knowledge, we are the first to use CF clinical isolates to address this question, and not only one, but eight different CF isolates of *S. aureus* were used [10]. Interestingly, the killing of none of the eight CF *S. aureus* isolates was impaired in CF PMNs in our study, only the killing of the reference strain, USA300 [10], indicating the importance of testing several CF isolates of *S. aureus*. We conclude that the topic remains controversial and additional studies are required.

The CF airway environment is a very complex inflammatory milieu that contains bacteria, immune cells, epithelial cells, mucins, exosomes, antibodies, DNA, NETs, cytokines, lipids, and soluble molecules of both host and microbial origin. PMNs transmigrating to the airways enter this hostile environment in CF. CF airway PMNs have a reduced ability to produce ROS compared to matched PMNs [48]. Studies comparing CF blood and airway PMNs described functional and signaling changes during airway homing, including increased surface expression of CD11b, CD66b, and CD63, and decreased expression of CD16 and CD14, important phagocytic receptors [14]. It is very challenging, though, to reliably purify CF airway PMNs without activation, and flow cytometry remains the only main method allowing for their studies. To perform more biologically meaningful studies (beyond surface marker exploration), several groups developed in vitro models where PMNs develop a CF airway-like phenotype [10,49,50,51].

Our CF sputum model was previously established to mimic the CF airway environment which included exposing healthy PMNs to pooled supernatants of CF sputum samples [10]. This study is the first to demonstrate the effect of the CF sputum on CF PMNs in which we show that it impairs the bacterial killing of MRSA (CF clinical isolates and USA300), which further confirms that our model mimics the conditions characteristic of the CF airway environment. We also further confirmed that the CF airway environment does not impair the phagocytosis of a CF clinical isolate of MRSA and there is no defect of *S. aureus* phagocytosis in CF PMNs. Another recent study also demonstrated that CF PMNs are not impaired in the phagocytosis of *S. aureus* [38]. However, our data showing that MRSA killing is significantly impaired following the addition of cytochalasin-B indicate that CF clinical isolates of MRSA are primarily killed through phagocytosis in healthy human PMNs. Cytochalasin-B is known to inhibit the uptake of bacteria, thus impairing phagocytosis [21,22]. Furthermore, it has also been shown to impair the translocation of granules containing MPO to the phagosome in human neutrophils, which also alludes to the impairment of phagolysosome fusion by the CF sputum as a possible explanation for impaired killing [21]. It has been well-established that phagosomal maturation, which includes the release of antimicrobial enzymes, ROS, and HOCl production, is crucial in killing *S. aureus* inside the phagosome of neutrophils [11,12,36,38,52,53,54]. H_2_O_2_ dismutates from superoxide anions produced by NADPH oxidase in the phagosome that reacts with MPO to generate compound I, which is a potent oxidant to mediate the electron oxidation of Cl^-^ to produce HOCl [55,56,57,58,59]. It is speculated that CF PMNs may be impaired in killing *S. aureus* in the phagosome due to the lack of normal CFTR function, which may affect chloride supply and hypochlorous acid production, impacting NADPH oxidase and MPO-chlorinating activities [39,40,55,60,61]. It was reported that CF PMNs have about 2.7-fold lower Cl^-^ in their phagosomes than normal PMNs, indicating that the production of HOCl is also lower [62]. However, CFTR is not the only chloride channel described on the phagosomal membrane that transports Cl^-^ in and out of the phagosome [59].

The phagosome matures when PMN granules fuse to the phagosomal membrane, transforming into the phagolysosome. Phagolysosomes are microbicidal vesicles that produce ROS through the assembled NADPH oxidase complex and contain proteases, lysozymes, and lipases [63,64]. NE, MPO, and CatD are contained in primary (azurophilic) granules that fuse with the phagosome membrane during phagolysosome fusion. In this study, we examined the co-localization of NE, MPO, and CatD with the phagosome of CF-sputum-treated PMNs after MRSA24 infection, in which the presence of these proteins illustrates that phagolysosome fusion occurred to enable their release inside the phagosome to co-localize with MRSA. NE and MPO activities are found in the CF sputum at high quantities, confirming that PMNs release these enzymes in CF [59,60]. The co-localization of NE with MRSA in the phagosome was not impaired by the CF sputum, however, that with MPO or CatD was.

Additionally, we discovered that phagosomal ROS was impaired during MRSA infection in PMNs. It has been previously reported that CF clinical isolates of *S. aureus* express high levels of superoxide dismutases which aid in the protection against human PMNs by neutralizing superoxide anions [65]. The differences in superoxide dismutase activities among the isolates could be partially responsible for the differences in their susceptibility to PMN killing and also for differences seen by the CF sputum. The reduced phagosomal ROS in MRSA-infected PMNs exposed to the CF sputum may further explain the discrepancy between our results that highlight that NE co-localizing to MRSA in the phagosome is not impaired; however, MPO and CatD are impaired despite their presence in primary granules. It has been previously described that MPO and CatD release into the phagosome is dependent on ROS in PMNs [13,66,67,68]. Therefore, it is possible that the primary granules are fusing to the phagosome; however, MPO and CatD are awaiting signals from the phagosomal ROS to be released from the granules into the phagosome, whereas NE is not dependent on ROS for its release. Studies show that CF macrophages infected with USA300 have an upregulation of CD63 on the phagosome membrane and that healthy PMNs infected with MRSA had 97–98% CD63 enrichment on their phagosomes, indicating that following MRSA ingestion, primary granule–phagosome fusion occurs [69].

Despite the inhibition of MPO and CatD co-localization with MRSA inside the phagosome, we did not see an inhibition of MPO and CatD activity in CF-sputum-treated PMNs. Primary granules containing NE, MPO, and CatD are not only trafficked to the phagosome, but are trafficked to the nucleus to mediate NET extrusion. Also, we previously reported that NET release was not impaired in *S. aureus*-infected PMNs exposed to the CF sputum [10]. Therefore, we further investigated whether primary granule component trafficking to the nucleus was impaired by the CF sputum. We did not see impairment of NE or MPO co-localization with the nucleus in MRSA-infected PMNs by the CF sputum.

The study has its limitations, since it only utilized a limited number of healthy subjects and PwCF, and only one MRSA isolate was tested in several experiments. PMNs obtained from a larger number of healthy individuals and PwCF and more MRSA isolates are required to be studied in the future.

Overall, our study demonstrates a unique mechanism by which the CF airway environment compromises MRSA killing by PMNs. We describe that the impaired killing of CF clinical isolates of MRSA by CF PMNs is associated with the reduced co-localization of the bacterium with primary granule components, MPO, CatD, and ROS generation inside the phagosome. The data suggest a CF-sputum-dependent negative effect on phagosomal bacterial killing in PMNs during MRSA infection.

## Figures and Tables

**Table 1:** Comparison of CF MRSA isolates used in this study.

CF Isolate ID in.			MRSA	PwCF ID CFBR	Mec Type	Agr Type	Spa Type	Polysacch Production	Strains	Ref.
This Work	ref. [17]	ref. [18]								
MRSA24	CFBRSa24	CFBR_09	Yes	219	II	2	t002	None	CF isolates	[17,18]
MRSA25	CFBRSa25	CFBR_10	Yes	134	II	2	t777	Normal		
MRSA47	CFBRSa47	CFBR_16	Yes	105	II	2	t002	None		
MRSA74	CFBRSa74	CFBR_24	Yes	201	II	2	t002	None		
USA300	-	-	Yes	-	-	-	-	-	Ref. strain	*S. aureus* subsp. aureus (ATCC^®^BAA1717^TM^)

Note. People with Cystic Fibrosis (PWCF) identification numbers according to the Cystic Fibrosis biospecimen registry. Mec type refers to the methicillin resistance gene each clinical isolate of *S. aureus* encodes. “w” in “PwCF” in lower case.

## Data Availability

The data of this manuscript will be uploaded to the Dryad public database available to the public.
